# Executive functions and theory of mind across age: The role of cognitive flexibility in perspective-taking skill

**DOI:** 10.1192/j.eurpsy.2021.1103

**Published:** 2021-08-13

**Authors:** D. Galletta, A.I. Califano, A. Santoro

**Affiliations:** 1 Department Of Head-neck Care Unit Of Psychiatry And Psychology “federico Ii” University Hospital Naples, “Federico II” University Hospital Naples, Italy, Naples, Italy; 2 Sanitary Pole, LA FILANDA LARS, SARNO, Italy

**Keywords:** Executive functions, theory of mind, cognitive flexibility

## Abstract

**Introduction:**

Research has demonstrated that greater cognitive flexibility and perspective taking skills are associated with positive outcomes throughout the lifespan. Cognitive flexibility is a core component of executive function allowing us to control goal-directed behaviour and to face new and unexpected conditions in the environment. Perspective-taking or Theory of Mind (ToM) refers to the capacity to make inferences about and represent others’ point of view, mental states and intentions.

**Objectives:**

The aim of this study was to assess age-related effects on executive functions and the role of cognitive flexibility in perspective-taking skills.

**Methods:**

Two age groups (34-44 years and 45-55 years) were compared on a task-switching paradigm the MATeM neuropsychological software (Maria Grazia Inzaghi, 2019) and all participants completed the Edinburgh Handedness Inventory (Oldfield, 1971), the IRI Interpersonal Reactivity Index (Davis, 1980), the RMET Reading the Mind in the Eyes (Baron-Cohen, 2001) and the BIDR-6 Balanced Inventory of Desirable Responding (Paulhus, 1991).

**Results:**

suggested that increased age was associated with decreased set-shifting, perspective-taking, mindreading abilities and increased tendency to give overly positive answers (socially desirable responding). Furthermore, participants with reduced cognitive flexibility (higher switch cost) were less able to attribute mental states to others and to appreciate another person’s point of view.

**Conclusions:**

It can be argued that readiness to appropriately adjust one’s behaviour according to a changing environment is related to flexibly shift between conflicting psychological perspectives. Future research include training studies which would further our understanding of these relationships and allow more effective cognitive and social interventions.

